# A Case of Resection of the Elbow Joint

**Published:** 1884-02

**Authors:** W. H. Washburne

**Affiliations:** Florence, Wis.


### Article IV.

### A Case of Resection of the Elbow-Joint. By W. H. Washburne, m.d., Florence, Wis.

Wm. J. G., a healthy young Irishman, æt. 21 years, recently arrived in this country, and while engaged in operating a horse-power wood-sawing machine, brought his arm in contact with the revolving saw, on December 7, 1882.

Upon examination, it was ascertained that the saw had made a transverse wound upon the posterior aspect of the forearm, about one inch below the point of the elbow.

The ulna was badly shattered from the olecranon process to the extent of about two inches. The radius was divided immediately above the bicipital tuberosity. The ulnar nerve was severed, and all the soft parts badly contused and lacerated, as the saw was not revolving very rapidly. The brachial artery was not injured. There was not any alarming hæmorrhage. The patient reacted well from the shock of the accident.

Two hours after the accident, with the efficient aid and council of my friends Drs. C. A. Fortier and R. W. Odell, resection was performed. The upper three inches of the ulna was removed, together with the head and neck of the radius, the humerus being left intact.

The arm was then placed in a modified Hodgen's splint and elevated from the bed, and carbolized dressings applied (ʒi to ℥vi of olive oil).

Inflammatory action of a high degree soon appeared, the part swelled enormously, so that the sutures which brought the edges of the wound into apposition were torn asunder. Dec. 8, the evening pulse and temperature were respectively 112 and $101\frac{4}{5}$°. From this time to Dec. 12, the pulse ranged from 90 to 108, and the temperature from 100 to $101\frac{1}{2}$°. Dec. 12, the patient had a severe chill, followed by a rise of temperature, the thermometer in the mouth indicating $102\frac{1}{2}$°; pulse 108. Nausea and vomiting now commenced, every kind of aliment, and even water, being rejected. A few doses of carbolic acid, gr. $\frac{1}{2}$ in ℥ij of lime-water, relieved these symptoms. He was then put upon quinine in 5 gr. doses, repeated every three hours. The wound was now discharging a large quantity of sanious pus. Dec. 13, temperature reduced to 99½° at 9 A. M.; pulse 86. From Dec. 13 to Dec. 26, temperature ranged from 100° to 102°; the quinine continued in smaller doses. The wound still dressed with carbolized olive oil (ʒiij to Oj). Abscesses now began to appear both above and below the elbow joint.

Feb. 1, 1883. Wound nearly healed; one abscess, situated just above the elbow and to the inner aspect of the arm, still discharging pus. The arm was now taken out of the Hodgen's splint, and placed in an anterior jointed splint, and the wounds dressed with basilicon ointment. Patient very much emaciated by his long confinement to the bed and his great physical suffering.

March 1. Several small pieces of necrosed bone were removed from the arm during the month of February. Patient gaining health and strength.

May 1. Several more small pieces of necrosed bone have been removed, and the wounds are now all healed.

November 30, 1883. Upon examination to-day, I find the patient a good specimen of a healthy man. His injured arm is still gaining in strength; in size, it is considerably smaller than its fellow. The elbow is anchylosed in a slightly flexed position. The muscles of the forearm possess considerable power, so much so, that he can lift and carry a weight of nearly if not quite fifty pounds; he can hold ten pounds out at arm's length, and is able to handle an axe in chopping wood; in fact, he is now employed as "chore" man by one of our citizens, and is able to perform all the duties pertaining to that place except that of sawing wood. He has acted in the capacity of hostler during the past fall.

The destruction of the ulnar nerve is a bad feature in this case, causing the patient more or less trouble and inconvenience all the time. The hand has but slight power of resisting the cold, and the little finger in particular is very sensitive to the cold, being cold all the time. It is devoid of sensation, and several times it has been cut and contused without his being aware that it had suffered an injury, until his attention was attracted to it by the sight of blood or ecchymosed spots.

On the whole, this man has an exceedingly useful member, considering the character of the injury; one that is a vast improvement on a "stump."

